# Prion peptide-mediated calcium level alteration governs neuronal cell damage through AMPK-autophagy flux

**DOI:** 10.1186/s12964-020-00590-1

**Published:** 2020-07-11

**Authors:** Ji-Hong Moon, Sang-Youel Park

**Affiliations:** Biosafety Research Institute, College of Veterinary Medicine, Jeonbuk National University, Gobong ro, Iksan, Jeonbuk 54596 South Korea

**Keywords:** Calcium, Prion, Autophagy flux, Neurodegeneration, AMPK

## Abstract

**Background:**

The distinctive molecular structure of the prion protein, PrPsc, is established only in mammals with infectious prion diseases. Prion protein characterizes either the transmissible pathogen itself or a primary constituent of the disease. Our report suggested that prion protein-mediated neuronal cell death is triggered by the autophagy flux. However, the alteration of intracellular calcium levels, AMPK activity in prion models has not been described. This study is focused on the effect of the changes in intracellular calcium levels on AMPK/autophagy flux pathway and PrP (106–126)-induced neurotoxicity.

**Methods:**

Western blot and Immunocytochemistry was used to detect AMPK and autophagy-related protein expression. Flow cytometry and a TdT-mediated biotin-16-dUTP nick-end labeling (TUNEL) assay were used to detect the percentage of apoptotic cells. Calcium measurement was employed using fluo-4 by confocal microscope.

**Results:**

We examined the effect of calcium homeostasis alterations induced by human prion peptide on the autophagy flux in neuronal cells. Treatment with human prion peptide increased the intracellular calcium concentration and induced cell death in primary neurons as well as in a neuronal cell line. Using pharmacological inhibitors, we showed that the L-type calcium channel is involved in the cellular entry of calcium ions. Inhibition of calcium uptake prevented autophagic cell death and reduction in AMP-activated protein kinase (AMPK) activity induced by human prion peptide.

**Conclusion:**

Our data demonstrated that prion peptide-mediated calcium inflow plays a pivotal role in prion peptide-induced autophagic cell death, and reduction in AMPK activity in neurons. Altogether, our results suggest that calcium influx might play a critical role in neurodegenerative diseases, including prion diseases.

**Video Abstract**

## Background

Protein accumulation in the brain is related to the pathogenesis of various neurodegenerative diseases, such as Parkinson’s disease, Alzheimer’s disease, as well as prion diseases [1]. Although prion disease is intermittent, it is lethal due to the non-availability of medical treatments to stop or interrupt the disease progression. Furthermore, prion transmission is a dangerous menace to community health due to the high resistance of prion proteins to standard disinfection methods [2]. The normal cellular prion protein (PrPc) is converted to scrapie-associated prion protein (PrPsc) through progressive changes involving the conversion of its α-helical domains to β-sheets [3]. These diseases share a general mechanism that includes structural modification of the disease-causing protein, leading to the generation of self-replicating particles and subsequent pathogenesis within the central nervous system (CNS) [4]. However, it remains unclear whether scrapie pathogenesis associated neurotoxicity is through PrPc or other mechanisms [5]. Human prion peptide corresponding to amino acid residues 106–126, can form fibrils in vitro and is noxious to hippocampal neurons [6]. It might be possible that the toxic form of PrP is created directly from PrPc or as a predecessor to pathological PrP [7].

Prion diseases are correlated with the deregulation of autophagy flux, as evidenced by the deposition of massive autophagic vacuoles in a hamster model of scrapie [8]. These autophagic vacuoles often enlarge in size and increase in number as neurons age, ultimately occupying the whole neurites [9]. Autophagic cell death, also known as programmed cell death II, is involved in the intracellular process that results in the dilapidation of cytosolic constituents inside the lysosomes [10]. The function of the lysosome is well-characterized in programmed cell death, although its exact role remains blurred [11]. Recent studies investigating the degradation of PrPsc revealed that the lysosome performs an imperative role in prion protein degradation and autophagy flux may be the main mechanism of PrPsc transport to the lysosomes [12–14]. Based on these observations, the authors suggested that autophagy flux might be beneficial in various diseases. Xu et al. advocated that the macro-autophagic system is stimulated in the scrapie-infected animals and human prion diseases [15]. ULK1, an autophagy protein, is necessary for cell survival during nutrient deficiency and is phosphorylated by AMPK [16].

AMP-activated protein kinase (AMPK) has been considered as a therapeutic target for several neurodegenerative diseases [17]. AMPK plays an essential role in preserving energy balance by phosphorylating and subsequently inactivating key enzymes involved in various biosynthetic pathways [18–20]. AMPK functions as a protein kinase that senses metabolic stress due to ATP depletion [21]. AMPK can be activated through phosphorylation by a tumor suppressor kinase, LKB1, under energy-deficient conditions and by CaMKK-β in response to depletion of intracellular calcium [22–24]. CaMKK activates AMPK in an AMP-independent manner and is itself activated by an increase in the intracellular calcium levels [25]. Thus, calcium might play an essential role in the activation of AMPK. Vitamin D3 improves the concentration of cytosolic calcium ions and generates autophagy flux [26, 27]. Maria et al. proposed an increase in the concentration of intracellular calcium induces autophagy flux, which is dependent on the autophagy-related genes (Atg) and CaMKK- β [28].

**C**alcium ions (Ca^2+^) are secondary messengers that play important roles in various signal transduction processes [29]. In healthy neurons, Ca^2+^-related cascades control numerous cellular functions, including gene transcription, exocytosis, intracellular respiration, and membrane trafficking [30]. Perturbation of calcium-regulating mechanisms results in extreme intracellular Ca^2+^, thereby triggering cell death [31]. Several reports suggest that PrP (106–126) alters calcium homeostasis through L-type calcium channels impairment [32, 33]. However, the molecular mechanisms regulating apoptosis during prion-mediated calcium influx remains largely unexplored.

Our previous study showed that prion protein-mediated neuronal cell death is triggered by the autophagy flux [34]. However, the relationship between intracellular calcium levels, AMPK activity, and autophagy flux has not been described yet. This study is focused on the effect of the changes in intracellular calcium levels on AMPK/autophagy flux pathway and PrP (106–126)-induced neurotoxicity. Our results suggest that preventing the calcium entry into the neurons affect AMPK/autophagy flux pathway and attenuates prion protein-mediated neurotoxicity. These outcomes suggest that regulating intracellular calcium levels, AMPK activity, and autophagy flux may be a practical therapeutic approach for neurodegenerative disorders including prion disease.

## Methods

### Cell culture

The primary murine cortex neuronal cells were prepared from embryonic 18-day ICR mice. Briefly, the brain was dissected in Hanks Buffered Saline Solution without Mg^2+^ and Ca^2+^ (HBSS: GIBCO, Grand Island, NY, USA), and digested in 0.25% trypsin containing DNAse I (2000 Units/mg) (GIBCO, Carlsbad, CA, USA) for 30 min at 37 °C. The obtained cell was diluted in fetal bovine serum (FBS), and cultured with Neuro basal media containing B-27 in tissue culture flasks coated with 50 μg/ml poly-D-lysine at a density of 3–4 × 10^5^ cells/cm^2^. Human neuroblastoma cell line SK-N-SH was obtained from the American Type Culture Collection (ATCC, Rockville, MD, USA). SK-N-SH cells were cultured in Minimum Essential Medium (Hyclone Laboratories, Logan, UT, USA) containing 10% FBS (GIBCO, Grand Island, NY, USA) and gentamycin (0.1 mg/mL) in a humidified incubator at 37 °C with 5% CO_2_. SK-N-SH (human neuroblastoma cell line, passage no.14) was acquired from the American Type Culture Collection (ATCC, Rockville, MD, USA).

### PrP (106–126) treatment

Synthetic prion peptides PrP (106–126) (sequence, Lys-Thr-Asn-Met-Lys-His-Met-Ala-Gly-Ala-Ala-Ala-Ala-Gly-Ala-Val-Val-Gly-Gly-Leu-Gly) were synthesized by Peptron (Seoul, Korea). The peptides were dissolved in sterile dimethyl sulfoxide (DMSO) at a concentration of 10 mM (stock) and stored at − 20 °C.

### Annexin V assay

Cells in the logarithmic phase were collected and cultured in 24-well plate at 4 × 10^4^ cells/well. Cell survival was evaluated using an annexin V Assay kit (Santa Cruz Biotechnology, CA, USA) following to the manufacturer’s procedure. The fluorescence was determined at 488-nm excitation and 525/30 emission using a Guava EasyCyte HT System (Millipore, Bedford, MA, USA).

### Lactate dehydrogenase assay

Cytotoxicity was measured by evaluating the levels of lactate dehydrogenase in the cell culture supernatant using the lactate dehydrogenase (LDH) detection kit (Takara Bio, Inc., Tokyo, Japan) following the manufacturer’s protocol. LDH activity was detected by measuring absorbance at 490 nm using a microplate reader (Spectra Max M2, Molecular Devices, Sunnyvale, CA, USA).

### BacMam transduction

GFP-tagged wild-type or mutant LC3B was overexpressed in neuronal cells via viral transduction using Premo Autophagy Sensor LC3B-GFP (BacMam 2.0) kit (Life Technologies, P36235). Briefly, LC3B-FP or LC3B (G120A)-FP viral particles (MOI = 30) were added to the growth medium and autophagosomes dynamics were visualized using fluorescence microscopy. The mutant LC3B (G120A)-FP was used as negative control.

### Immunocytochemistry

Cells in the logarithmic phase were collected and cultured in 1% gelatin-coated cover-slit (12 mm; Nalge Nunc International, Naperville, IL) in 24-well plate at 4 × 10^4^ cells/well. After treatment, fixed with 4% PFA in Phosphate Buffered Saline for 20 min at room temperature (RT). Cells were washed in sterilized Tris-Buffered Saline with 0.1% Tween 20 (TBST) for 10 min, then blocked for 15 min in TBST with 5% FBS, and then incubated for 3 h at RT with the primary antibodies (anti-phospho-AMPKα diluted 1:100; #2535, Cell Signaling Technology) diluted with in TBST with 5% FBS. Alexa Fluor 488-labeled donkey anti-rabbit IgG antibody (Molecular Probes, A21206) diluted 1:1000 was employed to visualize channel expression using fluorescence microscopy (Nikon Eclipse 80i). Image was evaluated using the NIS-Elements F ver4.60 Imaging software.

### Confocal microscopy

For confocal microscopy, the cells were cultured on coverslips. After treatment, the cells were fixed with 4% PFA (in PBS) for 20 min at room temperature (RT) and permeabilized using 0.3% Triton X-100 (in PBS) with 5% horse serum for 10 min. Subsequent incubations were performed in permeabilization buffer. The cells were incubated with primary antibodies for 60 min at RT, washed four times with PBS and incubated with Alexa Fluor 488, Alexa Fluor 568, and Alexa Fluor 647 conjugated secondary antibodies at a concentration of 0.3 μg/ml each for 60 min at RT. The coverslips were placed in mounting medium and imaged on a Zeiss LSM710 microscope equipped with a standard set of lasers through a 63× oil objective. Excitation wavelengths were 488, 543, and 633 nm. Bandpass filters were set at 500–550 (Alexa Fluor 488), 560–615 nm (Cy3, Alexa Fluor 568) and 650–750 nm (Alexa Fluor 647). Image acquisition was accomplished at the 12-bit rate. Settings were optimized to ensure suitable dynamic range, low background, and appropriate signal/noise ratio.

### Measurement of Ca^2+^ ions

Neuronal cells cultured on collagen-coated confocal dishes were incubated with 5 μM Fluo-4 AM (Invitrogen, Thermo Fisher Scientific) in culture media containing 1% FBS at 37 °C for 40 min. The cells were washed three times with HBSS (Hank’s Balanced Salt Solution). Intracellular calcium dynamics were visualized using a confocal microscope (Zeiss) with 488 nm excitation and 530 nm emission. For [Ca^2+^]*i* calculation, the method of Tsien et al. [35] was employed with the following equation: [Ca^2+^]*i* = Kd(F − Fmin) / (Fmax − F), where Kd is 345 nM for Fluo-4, and F is the observed fluorescence level. Each tracing was calibrated for the maximal (Fmax) by adding ionomycin (2 μM) and for the minimum intensity (Fmin) by adding EGTA (5 mM) at the end of each measurement.

### Western blot analysis

Cells in the logarithmic phase were collected and cultured in 6-well plate at 3 × 10^5^ cells/well. After treatments, cells were washed with PBS and lysed in lysis buffer [25 mM HEPES (4-(2-hydroxyethyl)-1-piperazineethanesulfonic acid), pH 7.4, 100 mM NaCl, 1 mM ethylene diamine tetra acetic acid (EDTA), 5 mM MgCl_2_, 0.1 mM dithiothreitol (DTT), and a protease inhibitor mixture]. Equal quantities of cellular proteins (more than 15 μg/μl) were electrophoretically resolved on a 10% sodium dodecyl sulfate (SDS) poly-acrylamide gel and transferred to a nitrocellulose membrane. Immuno-reactivity was detected through consecutive incubation with blocking solution using 5% skim milk and primary antibodies, followed by the corresponding horseradish peroxidase-conjugated secondary antibodies, and finally developed using enhanced chemi-luminescence substances i.e. west save gold detection kit (LF-QC0103, AbFrontier Inc.). The primary antibodies that anti-phospho-AMPKα diluted 1:1000 (#2535, Cell Signaling Technology), anti-AMPKα diluted 1:1000 (#2532, Cell Signaling Technology), anti-LC3B diluted 1:1000 (#4108, Cell Signaling Technology), anti-P62 diluted 1:1000 (#5114, Cell Signaling Technology) and anti-β-actin diluted 1:5000 (A5441, Sigma Aldrich) using antibody solution (1% skim milk in TBST) were used for immunoblotting. Images were inspected using a Fusion FX7 imaging system (Vilber Lourmat, Torcy Z.I. Sud, France). Densitometry of the signal bands was evaluated using the Bio-1D software (Vilber Lourmat, Marne La Vallee, France).

### TEM (transmission electron microscopy) analysis

Cells were fixed in 2% glutaraldehyde (Electron Microscopy Sciences, Hatfield, PA, USA) and 2% paraformaldehyde (EMS, USA) in 0.05 M sodium cacodylate (pH 7.2; Electron Microscopy Sciences) for 2 h at 4 °C. After, the samples were fixed in 1% osmium tetroxide (Electron Microscopy Sciences) for 1 h at 4 °C, dehydrated by incubating in alcohol solutions of increasing concentration (25, 50, 70, 90 and 100%) for 5 min each and entrenched in epoxy resin (Embed 812; Electron Microscopy Sciences) for 48 h at 60 °C following the manufacturers’ instructions. Ultrathin sections (60 nm) were cut using the LKB-III ultratome (Leica Microsystems GmbH, Wetzlar, Germany) and stained using 0.5% uranyl acetate (Electron Microscopy Sciences) for 20 min and 0.1% lead citrate (Electron Microscopy Sciences) for 7 min at room temperature. Images were captured on a Hitachi H7650 electron microscope (Hitachi, Ltd., Tokyo, Japan; magnification, 10,000×) at the Center for University-Wide Research Facilities (CURF), Chonbuk National University.

### Statistical analysis

Results are presented as the mean of replicas ± SEM. The Welch’s correction was employed for comparing two samples. The one-way ANOVA followed by the Tukey *post-hoc* test was applied for comparing multiple samples. All statistical analyses were implemented with GraphPad Prism version 5.0 software. P values such as * *p* < 0.05, ** *p* < 0.01 or *** *p* < 0.001 were considered statistically significant.

## Results

### Prion peptide increases intracellular Ca^2+^ and induces neuronal cell death

**Fig. 1:**
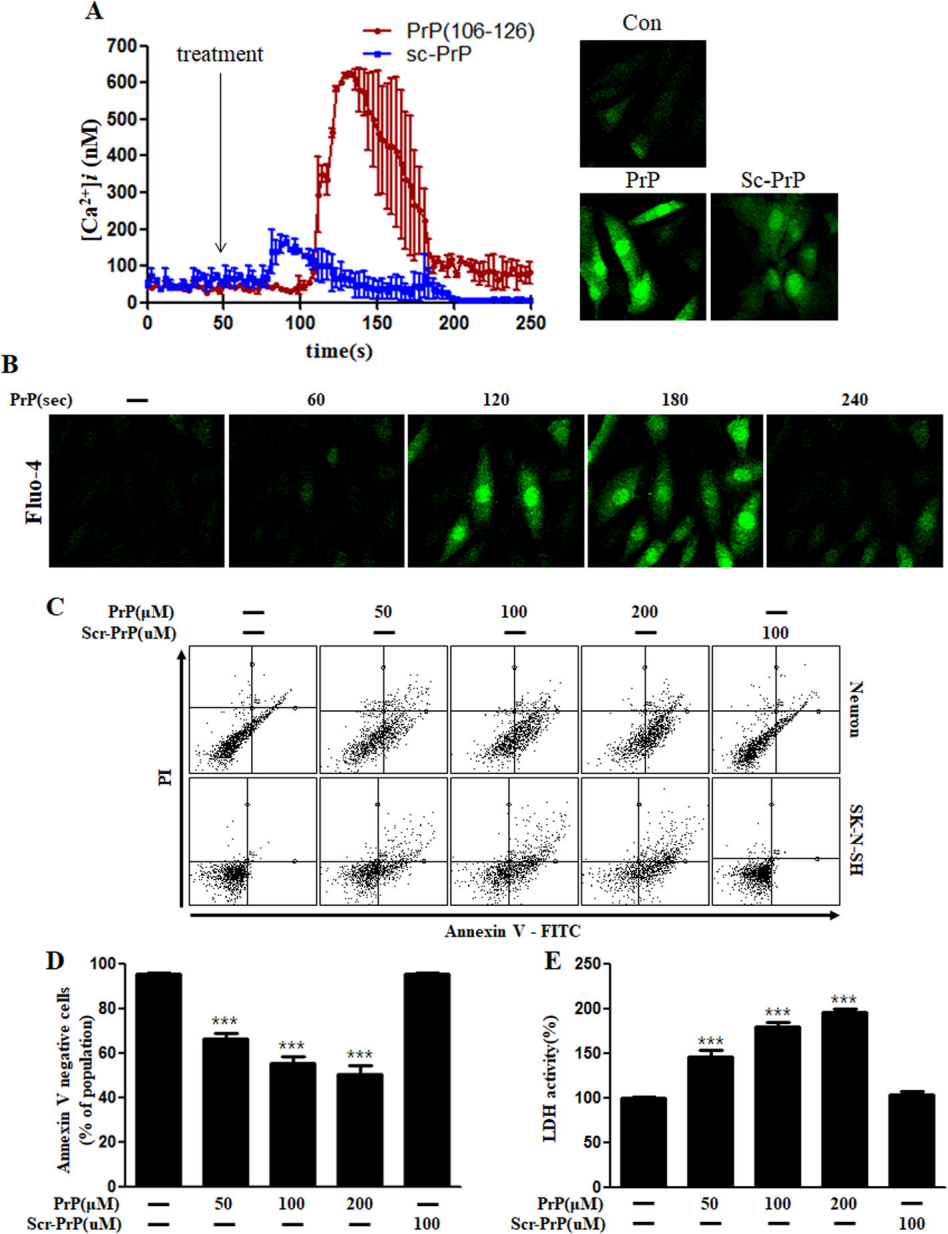
PrP (106–126) increased the intracellular Ca^2+^ levels and induced apoptosis in neurons. **a** Neuroblastoma cells (SK-N-SH) were exposed to fluo-4 AM and the changes in the intracellular Ca^2+^ levels were evaluated using confocal microscopy. The time point of PrP (106–126) or scrambled-PrP (106–126) treatments (100 μM) is shown by arrows. [Ca^2+^]*i* was measured at 200 s after the treatment in three independent experiments, indicate that, average kinetics of Ca^2+^ in the PrP groups more than the sc-PrP groups. Data are represented as mean ± SEM. **b** Green fluorescence (fluo-4) intensity that represents intracellular calcium concentration, changes time-dependently in SK-N-SH cells. **c** Primary neurons and SK-N-SH cells were exposed to different doses PrP (106–126) for 24 h. Cell viability was determined by Annexin V assay using FITC-annexin V, which binds to phosphatidylserine of the plasma membrane during the apoptotic process. **d** The bar graph represents the average number of annexin V negative cells. **e** LDH (lactate dehydrogenase) assay was performed to measure the LDH released into the culture medium. The results represent at least three independent experiments. Data are expressed as the mean ± SEM. *** *p* < 0.001, compared to control (one-way ANOVA). PrP, Prion peptide (106–126); sc-PrP, scrambled Prion peptide

We examined the role of prion peptide on intracellular Ca^2+^ levels in neuroblastoma (SK-N-SH) cells. PrP (106–126) treatment rapidly increased the intracellular Ca^2+^ levels in the cultured cells, although the increase was transient (Fig. 1a, b). Treatment with scrambled prion peptide showed a small increase in the intracellular Ca^2+^ level compared to prion peptide treatment. We investigated whether prion peptide induces neuronal apoptosis using Annexin V assay. PrP (106–126) exposure to both cultured primary neuronal cells as well as a neuroblastoma cell line (SK-N-SH) for 24 h resulted in cell death (Fig. 1c, d), whereas scrambled PrP (106–126) treatment did not induce neuronal cell death. Consistent with this, the amount of LDH released in the culture medium increased dose-dependently in PrP treated cells; scrambled PrP treated cells showed no change (Fig. 1e). These results show that prion peptide (106–126) treatment increases intracellular calcium levels and induces neutotoxicity.

### Prion peptide promotes neuronal apoptosis by calcium influx through L-type calcium channels

**Fig. 2:**
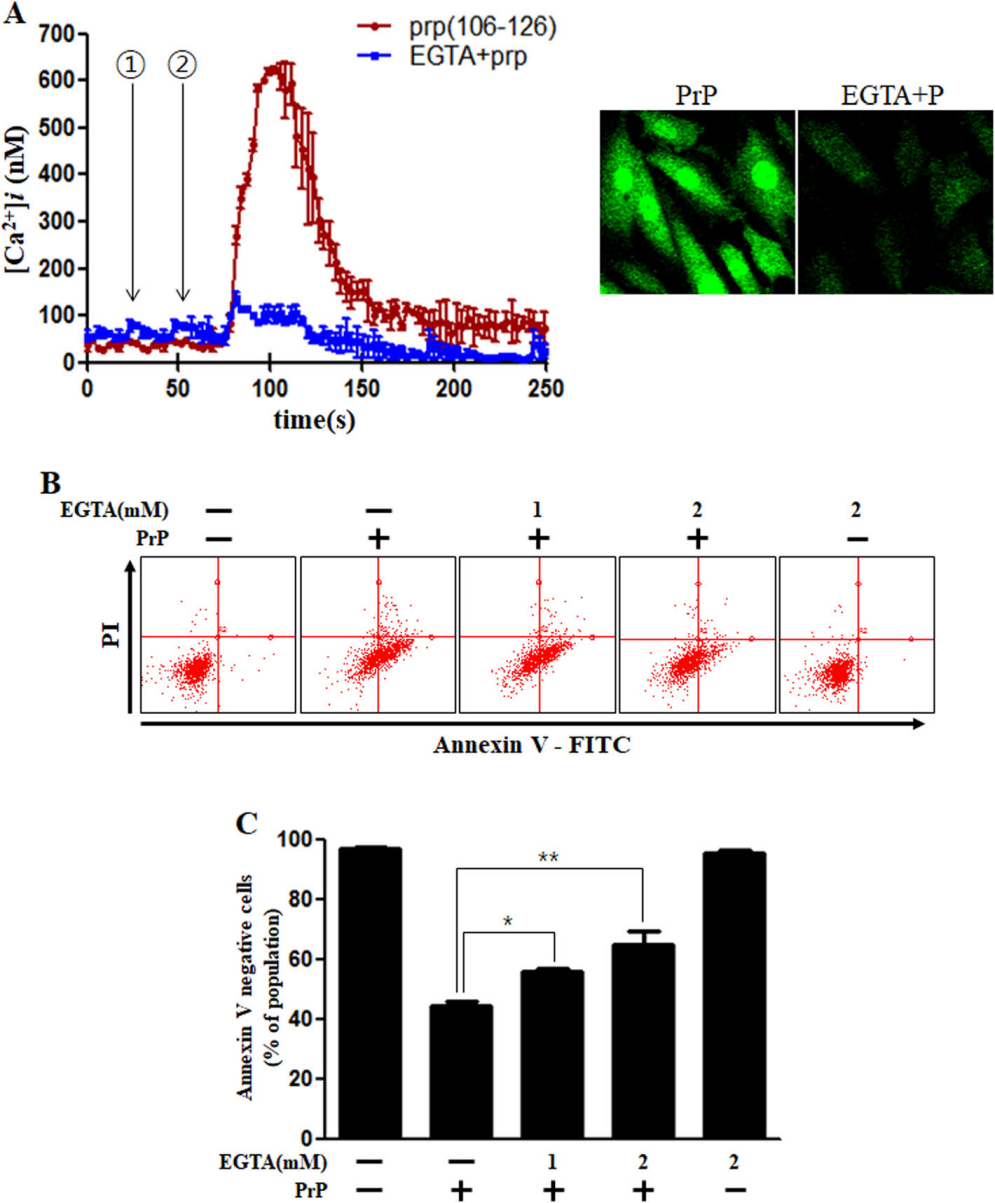
PrP (106–126)-mediated increase in the intracellular Ca^2+^ induced neuronal apoptosis. **a** SK-N-SH cells were treated with fluo-4 AM and the change in Ca^2+^ levels was measured using confocal microscopy. The time point of EGTA treatment (2 mM) is shown by the ① arrow and PrP (106–126) treatment (100 μM) is shown by the ② arrow. Data are represented as mean ± SEM. [Ca^2+^]*i* was measured at 200 s after the treatment in three independent experiments. **b** SK-N-SH cells were incubated with EGTA (a calcium chelator) for 1 h and then treated with 100 μM PrP (106–126) for 24 h. Cell viability was evaluated using FITC-annexin V, indicate that EGTA decreased PrP-mediated neurotoxicity. **c** The bar graph represents the average number of annexin V negative cells. The results represent at least three independent experiments. Data are expressed as the mean ± SEM. * *p* < 0.05, ** *p* < 0.01, compared to the PrP-treated group (one-way ANOVA). PrP, Prion peptide (106–126); EGTA, Egtazic acid

Studies suggest that prion peptide increases the intracellular calcium levels in several cell types [36–38]. Using EGTA, a useful calcium chelating agent in media, we investigated whether PrP (106–126)-mediated intracellular calcium increase is due to external influx or internal release from ER or mitochondria. Our results confirmed that the calcium present in the culture medium was taken up by the PrP (106–126) treated cells leading to an increase in the intracellular calcium since EGTA treatment repressed PrP (106–126)-mediated calcium increase (Fig. 2a) and neuronal apoptosis (Fig. 2b, c).

**Fig. 3:**
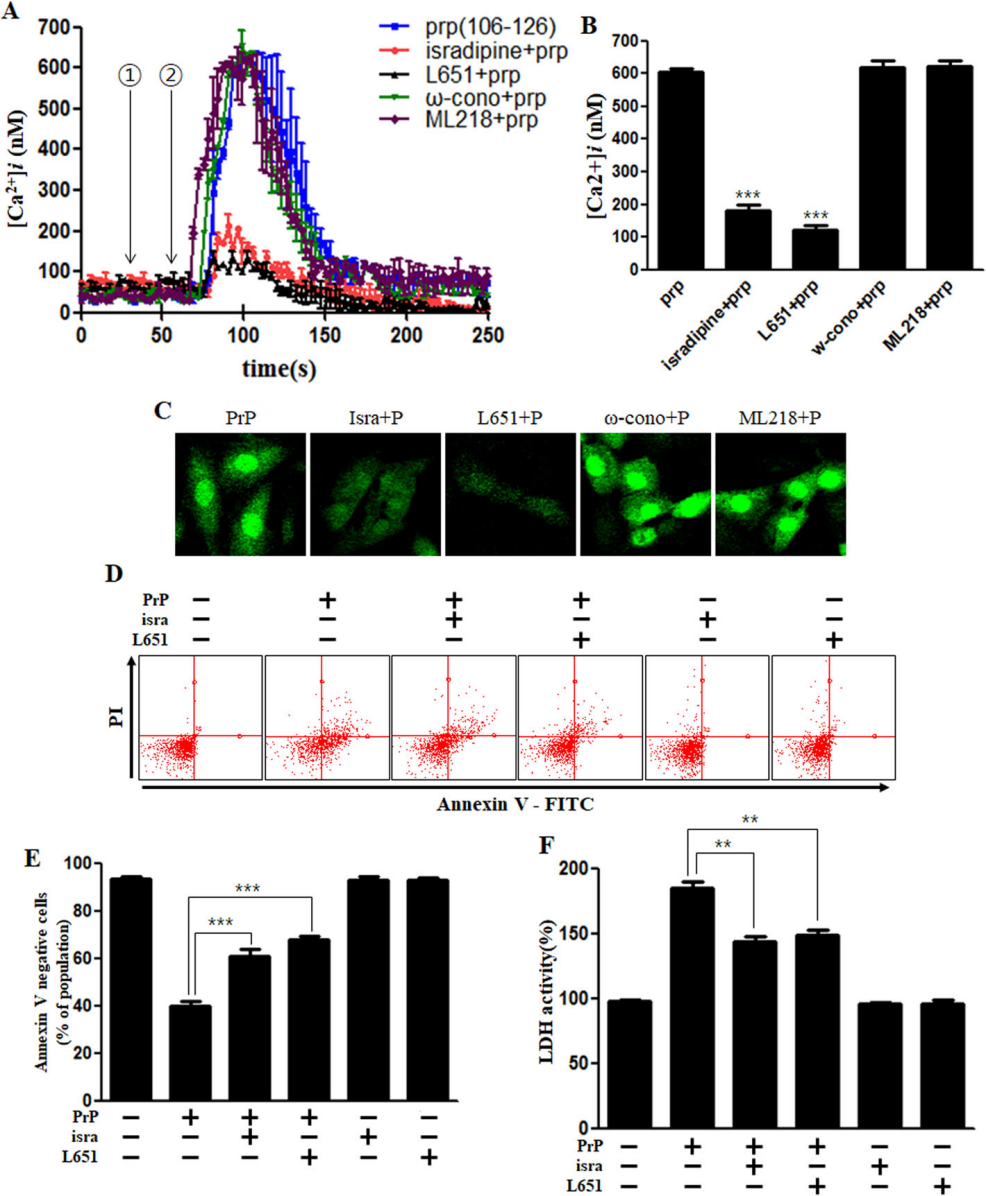
PrP (106–126)-mediated increase in intracellular Ca^2+^ occurs via L-type calcium channel. **a** SK-N-SH cells were exposed to fluo-4 AM and the alterations in Ca^2+^ levels were evaluated using confocal microscopy. The time point of isradipine (10 μM), L651,582 (20 μM) (L-type calcium channel blocker), ω-conotoxin GVIA (20 nM) (T-type calcium channel blocker) or ML218 (2 μM) (N-type calcium channel blocker) treatments is designated by the ① arrow and 100 μM of PrP (106–126) treatment is indicated by the ② arrow. Data are represented as mean ± SEM. [Ca^2+^]*i* was measured at 200 s after the treatment in three independent experiments. **b** The bar graph represents the average of the peak value of calcium levels. **c** Green fluorescence (fluo-4) intensity represents intracellular calcium concentration in SK-N-SH cells using confocal microscopy, indicate that L-type calcium channel blockers decreased PrP-mediated Ca^2+^ influx. **d** SK-N-SH cells were incubated with isradipine or L651,582 for 1 h and then exposed to PrP (106–126) (100 μM) for 24 h. Cell viability was assessed by annexin V assay using FITC-annexin V, indicate that isradipine and L651,582 decreased PrP-mediated neurotoxicity. **e** The bar graph represents the average number of annexin V negative cells. **f** LDH assay was performed to measure LDH released into the medium. Annexin V assay and LDH assay results represent at least three independent experiments. Data are expressed as the mean ± SEM. ** *p* < 0.01, *** *p* < 0.001; significant differences between each treatment group (Welch’s T-test). PrP, Prion peptide (106–126); isra, isradipine; ω-cono, ω-conotoxin GVIA

Silei et al. showed that L-type calcium channels are involved in this process [39]. We investigated the calcium channels involved in this process using various calcium channel blockers. We found that that treatment with isradipine and L651,582 the L-type calcium channel blockers obstructed the PrP-induced calcium increase (Fig. 3a, b). Fluorescence experiments also revealed that L-type calcium channel blockers repressed intracellular calcium increase (Fig. 3c). Moreover, the L-type calcium channel blockers attenuated prion peptide-induced neuronal apoptosis and LDH release (Fig. 3d, e, f). These results suggest that prion peptide-mediated increase in the intracellular calcium and neuronal cell death is via L-type calcium channels.

### Prion peptide induced neuronal apoptosis via AMPK down-regulation

**Fig. 4:**
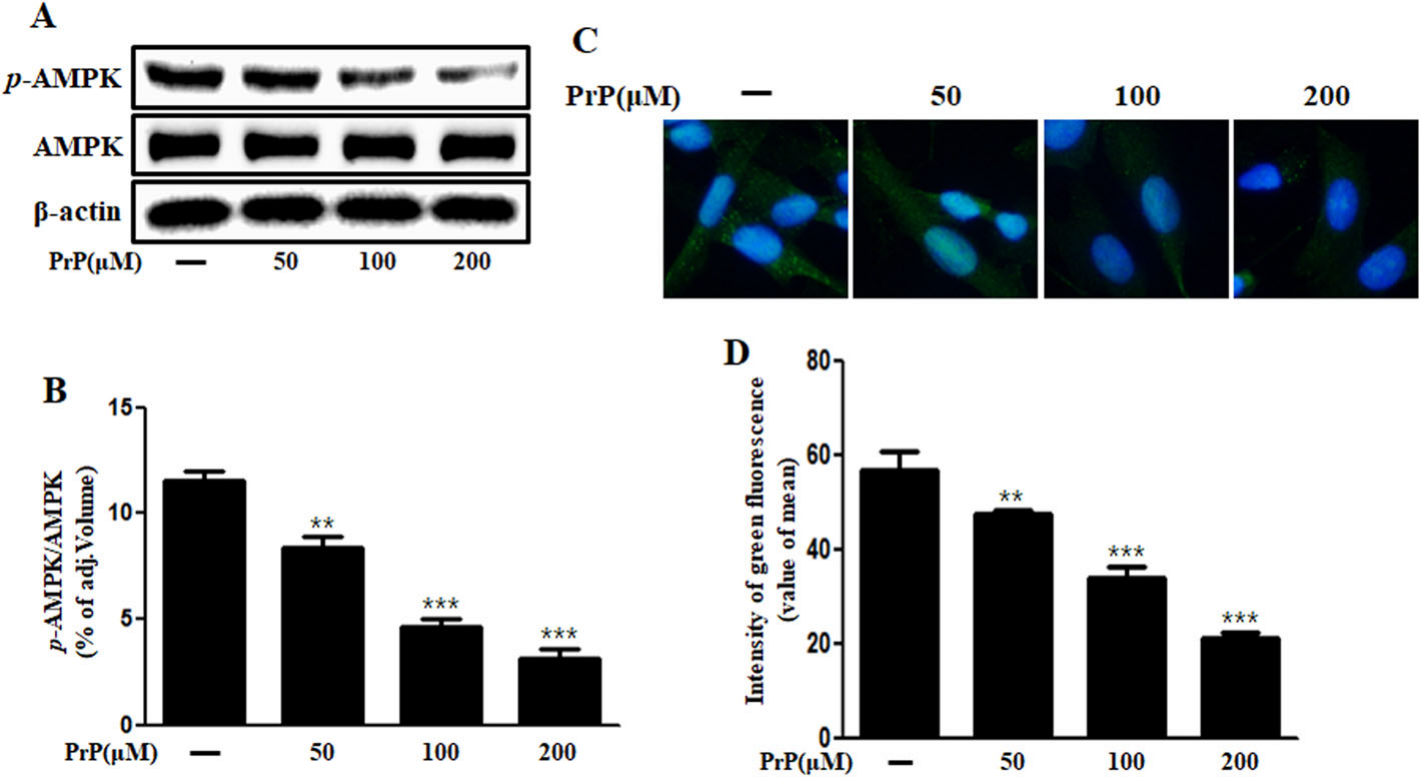
Prion peptide (106–126) treatment suppressed AMPK phosphorylation in neuronal cells. **a** Primary neurons were incubated with 50, 100, and 200 μM of PrP (106–126) for 6 h. Phospho-AMPK levels were analyzed by western blot analysis, indicate that PrP decreased AMPK phosphorylation. **b** Bar graph represents the mean levels of phospho-AMPK. **c** Immunocytochemistry to detect phospho-AMPK in SK-N-SH cells. **d** The bar graph represents the mean green fluorescence intensity. Results represent at least three independent experiments. Data are expressed as the mean ± SEM. ** *p* < 0.01, *** *p* < 0.001, compared to control (one-way ANOVA). PrP, Prion peptide (106–126); adj.volume, adjustment of volume (band volume minus background volume)

**Fig. 5:**
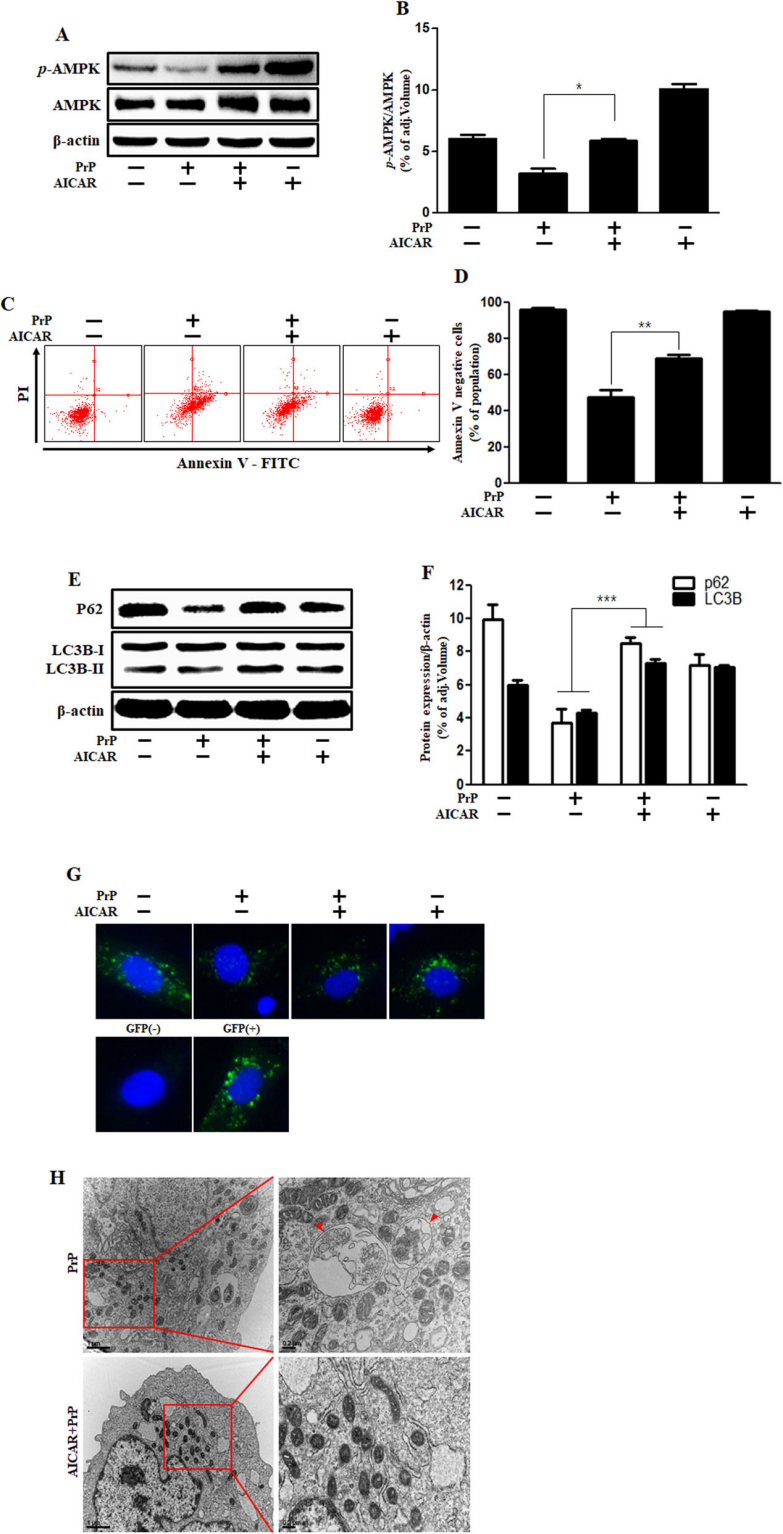
AMPK activator alleviates PrP (106–126)-induced autophagy flux and neurotoxicity. **a** Primary neuronal cells were incubated with AMPK activator (AICAR) for 1 h and then exposed to PrP (106–126) (100 μM) for 6 h. The cells were evaluated for *p*-AMPK levels by western blot analysis, indicate that AICAR increased p-AMPK. **b** The bar graph represents the average p-AMPK levels. **c** SK-N-SH cells were incubated with AICAR for 1 h and then treated to 100 μM of PrP (106–126) for 24 h. Cell viability was assessed by annexin V assay. **d** The bar graph represents the mean number of annexin V negative cells. **e** SK-N-SH cells were incubated with AICAR for 1 h and then exposed to PrP (106–126) (100 μM) for 6 h. P62 and LC3B expression were evaluated by western blot analysis, indicate that AICAR increased P62. **f** The bar graph represents the mean LC3B-II and p62 levels. **g** SK-N-SH cells were incubated GFP-LC3B virus (MOI 30) for over 18 h and then exposed to AICAR or PrP (106–126). Cell nuclei were stained with DAPI (blue) and evaluated by confocal microscopy. The cells were treated with negative and positive control reagent (CQ) at the same time. **h** SK-N-SH cells were incubated with AICAR for 1 h and then exposed to PrP (106–126) (100 μM) for 6 h. Autophagy flux mediated by AICAR and PrP (106–126) was analyzed by TEM. Arrowheads designate autophagosomes and autolysosomes. Western blot, Annexin V assay, and GFP-LC3B puncta formation assay results represent at least three independent experiments and TEM represents two independent experiments. Data are expressed as the mean ± SEM. ** *p* < 0.01, *** *p* < 0.001; significant differences between each treatment group (Welch’s T-test). PrP, Prion peptide (106–126); GFP (+), Positive control; GFP (−), Negative control; adj.volume, adjustment of volume (band volume minus background volume)

We observed that AMPK is involved in PrP (106–126)-mediated neuronal apoptosis. PrP (106–126) treatment inhibited AMPK phosphorylation in a dose-dependent manner (Fig. 4a, b) as assessed by immunohistochemistry (Fig. 4c, d). AMPK agonist, AICAR, increased the AMPK phosphorylation in the prion peptide treated cells (Fig. 5a, b). Further, AICAR treatment ameliorated PrP (106–126)-induced neuronal toxicity (Fig. 5c, d). In a previous study, we demonstrated that prion peptide generates autophagic flux in neuronal cells [34]. We examined the role of AMPK on prion peptide-mediated autophagy flux and found that AICAR treatment alleviated the prion peptide-induced autophagy flux activation (Fig. 5e, f). Furthermore, the GFP-LC3B puncta formation assay and TEM analysis verified the AICAR inhibited PrP (106–126)-induced autophagy flux activation (Fig. 5g, h). Altogether, our results show that prion peptide-mediated autophagy flux activation and neuronal apoptosis is regulated by AMPK. It also confirms that AMPK activation alleviates prion peptide-induced neuronal cell death through autophagy flux inhibition.

### L-type calcium channel blocker attenuated the prion peptide-mediated autophagy flux and phospho-AMPK reduction

**Fig. 6:**
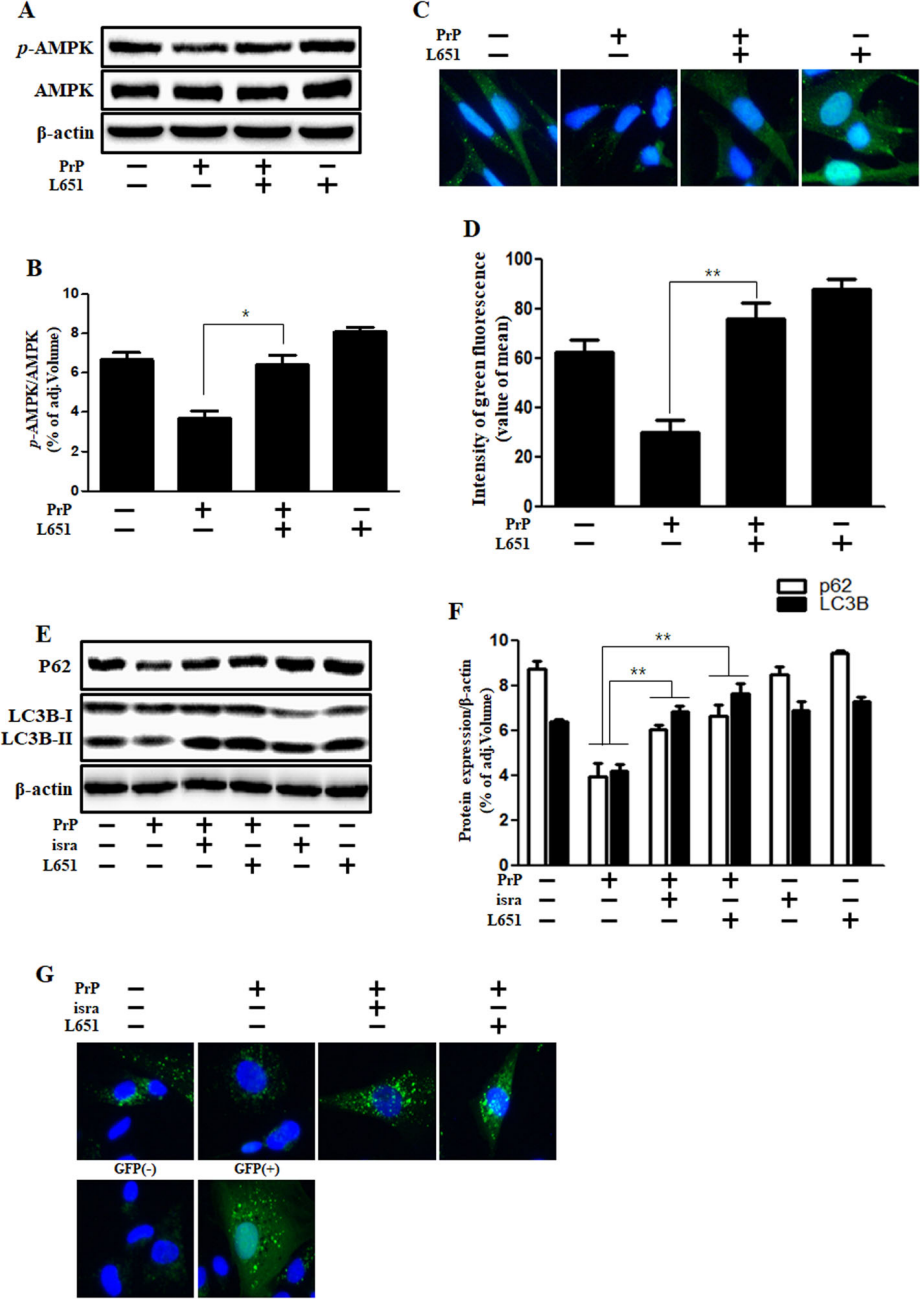
L-type calcium channel blocker reversed PrP (106–126)-mediated reduction in AMPK activity and increased autophagy flux in neuronal cells. **a** The primary neuronal cells were incubated with L651,582 for 1 h and then exposed to PrP (106–126) (100 μM) for 6 h. The treated cells were evaluated for *p*-AMPK by western blot analysis, indicate that L651,582 increased p-AMPK. **b** The bar graph represents the mean p-AMPK levels. **c** Immunocytochemistry for the *p*-AMPK was performed in SK-N-SH cells. **d** The bar graph represents the mean green fluorescence intensity. **e** Primary neuronal cells were incubated with isradipine and L651,582 for 1 h and then treated with PrP (106–126) (100 μM) for 6 h. The cells were evaluated for P62 and LC3B expression by western blot analysis, indicate that isradipine and L651,582 increased p62. **f** The bar graph represents the mean p62 and LC3B-II levels. **g** SK-N-SH cells were treated with GFP-LC3B virus for over 18 h and then exposed to PrP (106–126) for 6 h with isradipine or L651,582. Cell nuclei were stained with DAPI (blue) and evaluated by confocal microscopy. The cells were treated with negative and positive control reagent (CQ) at the same time. Western blot, immunocytochemistry, and GFP-LC3B puncta formation assay results represent at least three independent experiments. Data are expressed as the mean ± SEM. * *p* < 0.05, ** *p* < 0.01; significant differences between each treatment group (Welch’s T-test). PrP, Prion peptide (106–126); isra, isradipine; adj.volume, adjustment of volume (band volume minus background volume)

To investigate the effect of prion peptide-mediated changes in intracellular calcium levels on AMPK signaling and autophagy flux, we employed L651,582 an L-type calcium channel blocker. We found that L651,582 treatment improved the AMPK activity in PrP-treated neurons by western blot analysis (Fig. 6a, b) and immunocytochemistry (Fig. 6c, d). Further, L651,582 repressed the prion peptide-induced autophagy flux activation as assessed by western blot analysis (Fig. 6e, f) and immunocytochemistry (Fig. 6g). These results demonstrated that prion peptide-mediated suppression of AMPK activity and induction of autophagy flux are dependent on intracellular calcium levels. In conclusion, our results suggest that prion peptide changes intracellular calcium levels and autophagy flux through AMPK signaling in neuronal cells.

## Discussion

The role of autophagy in pathological process is under debate: inhibition of autophagy has been reported to have both beneficial and harmful effects for neurons [40–42]. autophagy has been recognized recently as a possible deleterious event as well. Activation of autophagic signaling was observed in ischemic brain [43], mediating ischemic neuronal death [44]. Jihoon et al. showed that autophagy flux in neurons is activated by amyloid β [45]. In previous study, we interpreted human prion peptide stimulates acute autophagy flux as autophagic cell death in neurons [34]. In our results, PrP (106–126) treatment induced LC3-II decrease and p62 decrease. This results could indicate autophagy flux inhibition. But we suggested the decrease of LC3-II and p62 are consequence of acute autophagy flux. Decreased p62/SQSTM1 levels are associated with autophagy activation and lysosomal degradation [46] and LC3-II is decreased after longer periods of autophagy activation [47]. After conducting other experiments such as GFP-LC3 puncta assay, TEM, ICC, we interpreted PrP (106–126) activates autophagy flux as autophagic cell death in neurons. There is another evidence that autophagy inhibitor (CQ) attenuated PrP (106–126)-induced autophagic cell death.

It is well known that AMPK and autophagy flux are the metabolic and cellular homeostasis regulators that are activated during starvation [48–50]. It has been well known that AMPK activation could trigger autophagy flux [51, 52]. However, our present study shows that prion peptide decreases AMPK phosphorylation while increases autophagic cell death (Fig. 5). We propose that prion peptide could cause AMPK dysfunction and induce autophagic cell death. The role of AMPK and autophagy flux in neurodegeneration remains unclear so that further studies are required.

Calcium plays a pivotal role in controlling the fate of neurons and targeting calcium channels might be a strategy for treating patients with neurodegenerative diseases [53]. Some studies showed that PrP (106–126)-mediated alteration in ER Ca^2+^ homeostasis is responsible for the increase in the cytosolic Ca^2+^ concentration, leading to caspase-3 activation, cytochrome *c* release, and apoptosis [36, 54, 55]. However, other studies suggest that scrapie or prion peptide cause a reduction in cytosolic calcium levels through L-type calcium channels by depolarizing K^+^ concentrations [33, 56, 57]. Jochen et al. claimed existence of PrPc is correlated with calcium influx through L-type calcium channels [58]. These inconsistent results may vary depending on the experimental method, condition or cell types. Although present studies suggest calcium ion as a secondary messenger, the experimental evidence for this has been very restricted. Various reports suggest that an increase in the intracellular Ca^2+^ levels stimulates autophagy flux via diverse signaling pathways such as mTOR, CaMKK, and AMPK [22, 24, 28]. We identified PrP (106–126) treatment triggered transitory rapid calcium influx, AMPK reduction and autophagic cell death through L-type calcium channels. Further studies are required to prove the role of calcium-dependent signaling proteins such as calcineurin, CaMKK, etc. in regulating autophagy flux. We postulate that prion peptide could be a beneficial tool to develop novel therapeutic strategies for prion diseases. Since we only studied the effects of prion peptide in-vitro, the intracellular calcium variation and AMPK activity in prion disease is yet to be established in-vivo. Further in-vivo studies are required to conclusively prove that the involvement of calcium-dependent pathways in prion peptide-induced neuronal apoptosis.

## Conclusions

Our results indicate that prion peptide increases intracellular calcium ion, resulting in decreased AMPK phosphorylation that stimulates autophagic cell death. Prion peptide causes an inflow of calcium ions through L-type calcium channels, which leads to neuronal injury through the AMPK/autophagic cell death signaling. Given that change in intracellular calcium levels, AMPK activity, and autophagy flux induce neurotoxicity, which has been observed in prion models, our results will improve the understanding of the prion disease pathogenesis and identifies L-type calcium channels as a potential therapeutic target.

## Data Availability

All data generated or analyzed during this study are included in this published article.

## Abbreviations

PrPsc: Scrapie isoform of the prion protein
PrPc: Normal prion protein
PrP: Prion protein
AMPK: AMP-activated protein kinase
CaMKK: Calmodulin-dependent protein kinase kinase
CNS: Central Nervous System
ULK1: Serine/threonine kinase Unc-51-like kinase-1
ATP: Adenosine triphosphate
LKB1: Liver kinase B1
Atg: Autophagy
EGTA: Ethylene Glycol Tetraacetic Acid
LDH: *Lactate dehydrogenase*
AICAR: *AMPK* agonist 5-aminoimidazole-4-carboxamide ribonucleotide
TEM: *Transmission electron microscopy*
ER: Endoplasmic reticulum
